# Parallel Image-Based Visual Servoing/Force Control of a Collaborative Delta Robot

**DOI:** 10.3389/fnbot.2022.922704

**Published:** 2022-06-01

**Authors:** Minglei Zhu, Cong Huang, Zhiqiang Qiu, Wei Zheng, Dawei Gong

**Affiliations:** ^1^School of Mechanical and Electrical Engineering, University of Electronic Science and Technology of China, Chengdu, China; ^2^Science and Technology on Thermal Energy and Power Laboratory, Wuhan Second Ship Design and Research Institute, Wuhan, China

**Keywords:** image-based visual servoing/force control, collaborative robot, Delta robot, trajectory tracking, image moment visual servoing

## Abstract

In this paper, a parallel Image-based visual servoing/force controller is developed in order to solve the interaction problem between the collaborative robot and the environment so that the robot can track the position trajectory and the desired force at the same time. This control methodology is based on the image-based visual servoing (IBVS) dynamic computed torque control and couples the force control feedback in parallel. Simulations are performed on a collaborative Delta robot and two types of image features are tested to determine which one is better for this parallel IBVS/force controller. The results show the efficiency of this controller.

## 1. Introduction

Recently, a large number of research studies focus on the study of collaborative robots in the domains of mechanical, sensing, planning, and control issues in order to improve the safety and dependability of robotic systems (Magrini and De Luca, 2016). When collaborative robots are performing tasks, such as picking and placing of objects, deburring, polishing, spraying, and high precision positioning assembly (Yang et al., 2008; Wu et al., 2017; Xu et al., 2017), the control of contact forces and the position are all necessary. When collaborative robots are interacted with the environment, the contact forces exist and should be controlled properly, otherwise, this force may damage the robot manipulator or the human operating the robot.

Compared with serial robots, parallel robots show better performance in terms of high speeds and accelerations, payload, stability, stiffness, and accuracy. Due to these advantages, parallel robots is widely applied as collaborative robots (Zabihifar and Yuschenko, 2018; Jeanneau et al., 2020; Fu et al., 2021). However, the force/position control of parallel robots has been rarely addressed since the structure of parallel robots is complex and the relationship between the input and output of the controller is highly non-linear (Merlet, 2006; Dai et al., 2021). When parallel robots are controlled with a classical model-based computed torque controller, the position accuracy relies on the model precision. Nevertheless, even a detailed robot model still can not guarantee high accuracy because of the errors from manufacturing, assembly, and external disturbances in practice. To overcome this problem, we need to find an alternative way to bypass the complex parallel robot architectures. Visual servoing is a type of an external sensor-based controller which takes one or several cameras as external sensors and closes the control loop with the vision feedback (Chaumette and Hutchinson, 2006; Chaumette et al., 2016; Fu et al., 2021; Zhu et al., 2022). With this controller, the pose of the robot can be directly estimated from the camera and we can bypass the complex calculation of the direct kinematic model. The visual servoing can be divided into three categories: image-based visual servoing (IBVS), position-based visual servoing (PBVS), and 2.5D visual servoing. Image-based visual servoing regulates the error directly in the image plane, and the position-based visual servoing takes the pose of the camera with respect to some reference coordinates frame to define the feature to be controlled and the entire control algorithm is realized in the Cartesian space. 2.5D visual servoing is a combination of IBVS and PBVS. Compared with PBVS and 2.5D visuals servoing, image-based visual servoing is realized in the image plane, so that it will not cause the loss of tracking target during the servoing. In addition, IBVS is more robust to the camera calibration errors and the noise from the camera observation.

Force control is important in the process when collaborative robots are operating tasks requiring interaction with the environment, such as contour-following operations, assembly of mechanical components, and the use of any mechanical tool. Several types of force control schemes have been proposed and they can be divided into two main categories: direct force control and indirect force control (Siciliano and Villani, 1999). Indirect force control includes the admittance control (Mason et al., 1981) and impedance control (Hogan, 1985). These two control methods are based on creating a model between the interaction forces and the pose of the manipulator and replacing the direct control of the contact force with the position control of the robot manipulator. The parameters of the impedance model or the admittance model, such as the stiffness, damping, and inertia, should be aware in advance and the performance of the controller is affected by the accuracy of these parameters. Direct force control includes the hybrid position/force control (Raibert and Craig, 1981) and parallel position/force control (Chiaverini and Sciavicco, 1993). The hybrid position/force controller is designed with two individual controllers: position controller and force controller. They are developed for each decoupled subspace and connected with a selection matrix. When hybrid position/force control is applied, the geometric description of the interaction environment ought to be available in advance. In addition, for one dedicated DOF, we cannot control the contact force and the position simultaneously. The parallel position/force controller combines the position control loop and the force control loop in parallel. It can reach the goal of controlling position and force at the same time (Chiaverini and Sciavicco, 1993) without the selection matrix. The dynamic parameters of the interaction model and the geometric description of the environment are not indispensable when we apply this method.

Therefore, in the current research, a novel controller: a parallel IBVS/force controller is created. The IBVS dynamic controller is applied to perform the position control and the force feedback is coupled in parallel. Two controlled variables, contact forces and the image features, are combined together to reach the goal of controlling the force and position directly in the image space at the same time. Considering the fact that the accuracy of position control directly affects the precision of contact force control, in this paper, two types of image features, normalized image points and image moments (Tahri and Chaumette, 2005; Dallej et al., 2006; Andreff et al., 2007), are selected as the vision feedback, and the comparison of controller performance will be tested in order to determine which one is better for the parallel IBVS/force control of parallel robots.

To the best of our knowledge, this is the first time that the parallel IBVS/force control is proposed and applied to the control of a collaborative Delta robot. The compensation of the robot vision dynamic non-linearities are considered and both position and force can be controlled in all directions. In addition, two types of IBVS with different image features, normalized image points and image moments, are tested to determine which one is more suitable for this controller.

This paper is organized as follows. In Section 2, the IBVS dynamic model is created and a simple recall on the normalized image point visual servoing and image moment visual servoing are given. In Section 3, the parallel IBVS/force control method is proposed and presented in detail, in addition, the stability analysis is given. Then, in Section 4, the way of creating the dynamic model of the Delta robot is presented. Simulations and the analysis of results are given in Section 5. In the end, we draw the conclusion in Section 6.

## 2. Image-Based Visual Servoing Dynamic Model

For image-based visual servoing, the vector of image features obtained from the camera observation is assigned to **s**. With the well-known interaction matrix **L**_*a*_ (Chaumette and Hutchinson, 2006), the IBVS kinematic model can be written as follows:


(1) $$\begin{array}{l} \dot{\text{s}}={\text{L}}_{a}\tau \end{array}$$

where **τ** is the camera-object kinematics screw.

When the dynamics of robots are considered, the first order of visual servoing is no longer sufficient because the velocity of the image features could not correspond to the acceleration of the robot. Then, we needed to find the relationship between the image features acceleration and the robot kinematics, and dynamics with a second-order model so that the computed torque controllers can be obtained directly in image space. This second-order visual servoing interaction model can be obtained by differentiation of Equation (1):


(2) $$\begin{array}{l} \ddot{\text{s}}={\text{L}}_{a}\frac{\text{d}}{\text{d}t}\tau +{\dot{\text{L}}}_{a}\tau \end{array}$$

In addition, the time derivation of the kinematic screw is as follows:


(3) $$\begin{array}{l} \frac{\text{d}}{\text{d}t}\tau =\text{a}+\left[\begin{array}{c} v\times w\\ 0 \end{array}\right] \end{array}$$

where **a** is the spatial relative camera-object acceleration screw. Equation (3) is obtained in the condition that the camera frame is not inertial with respect to the observed object.

Then (2) can be transformed to


(4) $$\begin{array}{l} \ddot{\text{s}}={\text{L}}_{s}\text{a}+{\text{L}}_{s}\left[\begin{array}{c} v\times w\\ 0 \end{array}\right]+\left[\begin{array}{c} \tau^{T}{\text{H}}_{1}\\ \cdots \\ \tau^{T}{\text{H}}_{n} \end{array}\right]\tau \end{array}$$

where ${\text{H}}_{i}≐\left(\frac{\partial l_{i}}{\partial \text{s}}\right){\text{L}}_{s}+\left(\frac{\partial l_{i}}{\partial z}\right){\text{L}}_{z}$, and the depth *z* between the camera and the object has the following property *ż* = **L**_*z*_**τ**.

After some simple manipulations, we can rewrite Equation (2) as follows:


(5) $$\begin{array}{l} \ddot{\text{s}}={\text{L}}_{a}\text{a}+{\text{L}}_{v} \end{array}$$

**L**_*v*_ corresponds to the differentiation of the interaction matrix and the Coriolis acceleration. It can be written in the form:


(6) $$\begin{array}{l} {\text{L}}_{v}=\left[\begin{array}{c} \tau^{T}\Omega_{x}\tau \\ \tau^{T}\Omega_{y}\tau \end{array}\right] \end{array}$$

**L**_*v*_ is a function of **s**, **τ**, and the depth **Z** between the object and the camera and the expression of Ω_*x*_ and Ω_*y*_ can be found in Mohebbi (2012).

The most common image feature of visual servoing is the normalized image points. For a given 3D point P in space and its coordinates ${\left[X_{p},Y_{p},Z_{p}\right]}^{T}$ with respect to the camera frame, we can get the normalized coordinate **s** = [*x, y*]^*T*^ = [*X*/*Z, Y*/*Z*]^*T*^. *Z* is the so-called vision depth between the object and the camera. The interaction matrix related to the image point feature **s**, **L**_*s*_ takes the following well-known form (Chaumette and Hutchinson, 2006):


(7) $$\begin{array}{l} {\text{L}}_{s}=\left[\begin{array}{cccccc} -\frac{1}{Z} & 0 & \frac{x}{Z} & xy & -x^{2}-1 & y\\ 0 & -\frac{1}{Z} & \frac{y}{Z} & y^{2}+1 & -xy & -x \end{array}\right] \end{array}$$

while the interaction matrix of a set of normalized image points can be obtained by vertically stacking the individual matrices.

With the development of image processing technology, another image feature can be applied and has been proven to be effective in image-based visual servoing is planar image moments (Chaumette, 2004). The target T to be observed can be a dense object defined by a set of closed contours or a discrete set of image points (Tahri and Chaumette, 2005). The 2D image moments *m*_*ij*_ of order *i* + *j* are defined by:


(8) $$\begin{array}{l} m_{ij}=\iint_{T}x^{i}y^{i}dxdy \end{array}$$

where *x* and *y* are the coordinates in the camera frame of any point *M* belonging to the object T. Several independent image moments can be calculated with this definition: the coordinates *x*_*g*_, *y*_*g*_ of the center of gravity of the target, the area *a* of the target, the orientation α, and the invariant image moments *c*_1_, *c*_2_ (see definition in Tahri and Chaumette, 2005). The expression of the interaction matrix **L**_*m*_*ij*__ of any image moment of order *i* + *j*, *m*_*ij*_ has been provided in Chaumette (2004). The differentiation of the interaction matrix and the Coriolis acceleration term **L**_*v*_ related to the image moment *m*_*ij*_ can be found in Fusco (2020).

## 3. Development of the Parallel IBVS/Force Controller

### 3.1. Design of the Parallel IBVS/Force Controller

The requirements of restricted motion tasks, which comprise simultaneous motion and force control, cannot be met by classical computed torque control applied in joint space. Therefore, we consider the general dynamic model of robots expressed in the task space.


(9) $$\begin{array}{l} {\text{M}}_{\chi }\left(\chi \right)\ddot{\chi }+{\text{C}}_{\chi }\left(\chi ,\dot{\chi }\right)+{\text{G}}_{\chi }\left(\chi \right)+\text{F}+{\text{J}}^{T}\left(\chi \right)f=\tau_{\chi } \end{array}$$

where χ is the set of Cartesian coordinates of the robots. **M**_χ_ is the inertia matrix in task space, ${\text{C}}_{\chi }\left(\chi ,\dot{\chi }\right)$ is the term Coriolis and centrifugal. **G**_χ_(χ) represents the gravity, and ***f*** is the vector of friction forces. **F** is the contact force caused by the robot manipulator and the environment. **τ** is the projection of the generalized torques at the joints in the task frame. **J** is the Jacobian matrix of the robot.

In the condition that the parameters of the robot model are perfectly known, then the parallel position/force control law can be written as follows:


(10) $$\begin{array}{l} \tau_{\chi }={\hat{\text{M}}}_{\chi }\left(\chi \right)u+{\hat{\text{C}}}_{\chi }\left(\chi ,\dot{\chi }\right)+{\hat{\text{G}}}_{\chi }\left(\chi \right)+\hat{\text{F}}+{\text{J}}^{T}\left(\chi \right)\hat{f} \end{array}$$

where ˆ represents the estimated value. We suppose that the position and force control loops are, respectively, a linear PD and PI controller, then we have:


(11) $$\begin{array}{l} \;u=u_{p}+u_{f}\\ u_{p}={\ddot{\chi }}^{d}+{\text{K}}_{v}\left({\dot{\chi }}^{d}-\dot{\chi }\right)+{\text{K}}_{p}\left(\chi^{d}-\chi \right)\\ u_{f}={\text{K}}_{f}\left({\text{F}}^{d}-\text{F}\right)+{\text{K}}_{i}\int_{0}^{t}\left({\text{F}}^{d}-\text{F}\right)d\tau \end{array}$$

where ()^*d*^ represents the desired value. **K**_*v*_, **K**_*p*_, **K**_*f*_, and **K**_*i*_ are positive diagonal matrices. From this control law, we see that because of the integral item, the position, and force in all degrees of freedom (DOF) with the force control loop taking precedence over the position control loop is considered.

**Figure 1:**
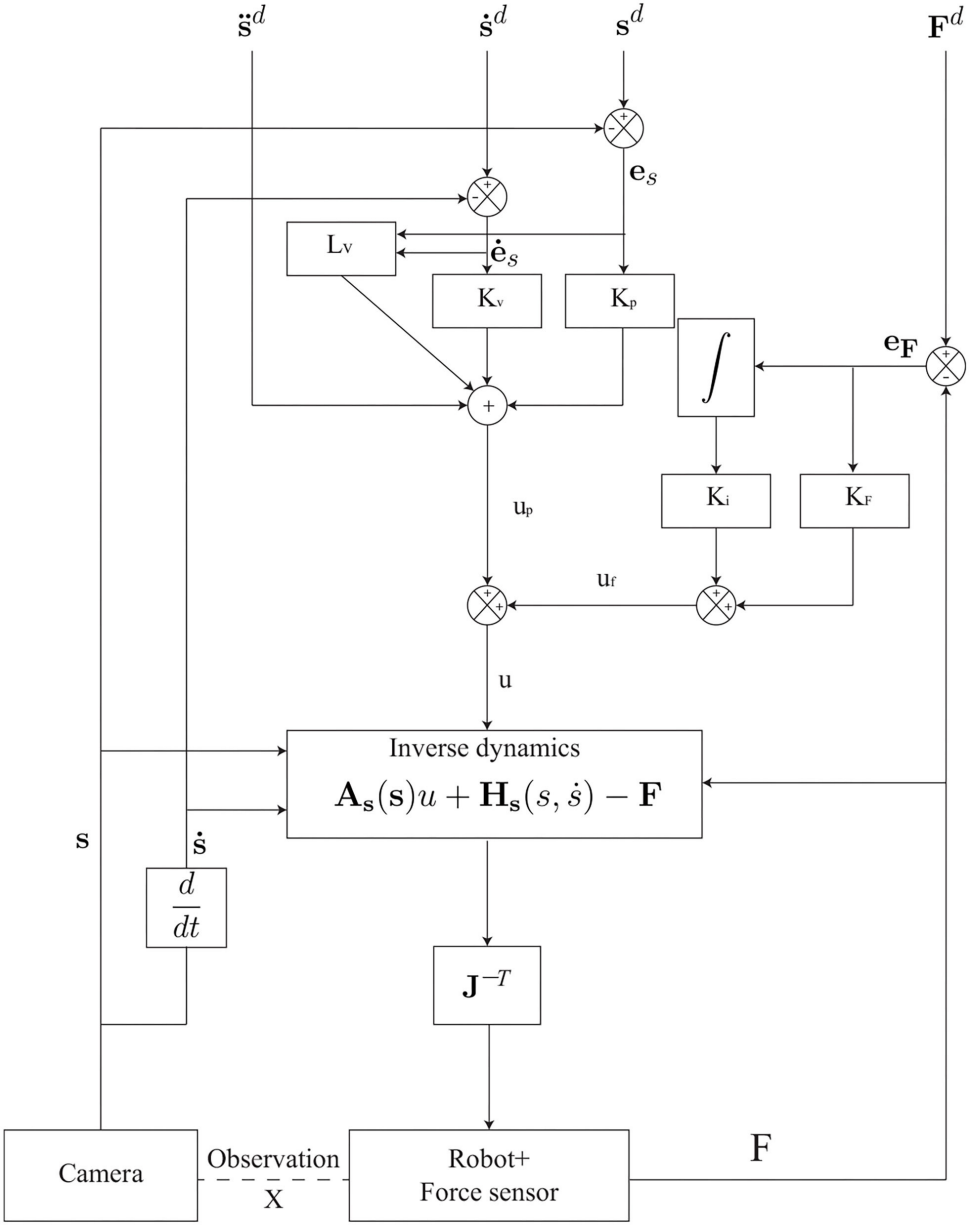
The parallel image-based visual servoing (IBVS)/force control scheme.

The drawback of applying the model-based position controller (with joint sensor) has been detailed and presented. Moreover, the combination of a sensor-based force control loop and a model-based control loop leads to a control architecture that is not homogeneous. Then, we replace the model-based position controller with the image-based visual servoing controller. The resultant control law applied to the robots in task space can be written as follows and the control scheme is illustrated in Figure 1.


(12) $$\begin{array}{l} \tau_{\chi }={\hat{\text{M}}}_{\chi }\left(\chi \right)[{\hat{\text{L}}}_{a}^{+}\left({\ddot{\text{s}}}^{d}+{\text{K}}_{v}{\dot{\text{e}}}_{\text{s}}+{\text{K}}_{p}{\text{e}}_{\text{s}}-{\text{L}}_{v}\right)+{\text{K}}_{f}{\text{e}}_{f}+\\ \;{\text{K}}_{i}\int_{0}^{t}{\text{e}}_{f}dt]+{\hat{\text{C}}}_{\chi }\left(\chi ,\dot{\chi }\right)+{\hat{\text{G}}}_{\chi }\left(\chi \right)+\hat{\text{F}}+{\text{J}}^{T}\left(\chi \right)\hat{f} \end{array}$$

where ${\ddot{\text{s}}}^{d}$ is, respectively, the desired acceleration of the image features, ${\text{e}}_{\text{s}}={\text{s}}^{d}-\text{s}$ is the error of image features, ${\dot{\text{e}}}_{\text{s}}$ is its time derivative, and ${\text{e}}_{f}={\text{F}}^{d}-\text{F}$ is the error of the force applied on the robot. The rest are the same as we defined in Equation (10).

### 3.2. Stability Analysis

The parallel IBVS/force control (12) aims at composing the compliant displacement caused by the contact force with the desired position. In order to get a clear description of the behavior during interaction with parallel control, it is necessary to consider the contact environment. We suppose the interaction environment is a planar surface, the rotation matrix of the compliant frame ∑_*c*_


(13) $$\begin{array}{l} {\text{R}}_{c}=\left[{\text{t}}_{1c}\;{\text{t}}_{2c}\;{\text{n}}_{c}\right] \end{array}$$

where **n**_*c*_ is normal and **t**_1*c*_, **t**_2*c*_ are tangential to the plane. The contact model is supposed to be a spring model, and the contact force can be calculated with ***f*** = **K**_*e*_(**P**_*e*_ − **P**_*o*_), **P**_*o*_ represents any position of the undeformed plane, and **P**_*e*_ is the equilibrium position. **K**_*e*_ denotes the contact stiffness matrix and can be obtained from


(14) $$\begin{array}{l} {\text{K}}_{e}={\text{R}}_{c}diag\left\{0,0,k_{fn}\right\}{\text{R}}_{c}^{T}=k_{f,n}{\text{n}}_{c}{\text{n}}_{c}^{T} \end{array}$$

with *k*_*fn*_ is positive.

The elastic spring model shows that the contact force is normal to the plane and the desired force **F**_*d*_ is aligned with **n**_*c*_.

The equilibrium position can be described as


(15) $$\begin{array}{l} {\text{P}}_{e,\infty }=\left(\text{I}-{\text{n}}_{c}{\text{n}}_{c}^{T}\right){\text{P}}_{d}+{\text{n}}_{c}{\text{n}}_{c}^{T}\left(k_{f,n}^{-1}{\text{F}}_{d}+{\text{P}}_{o}\right)\\ {\text{F}}_{\infty }=k_{f,n}{\text{n}}_{c}{\text{n}}_{c}^{T}\left({\text{P}}_{e,\infty }-{\text{P}}_{o}\right)={\text{F}}_{d} \end{array}$$

**Figure 2:**
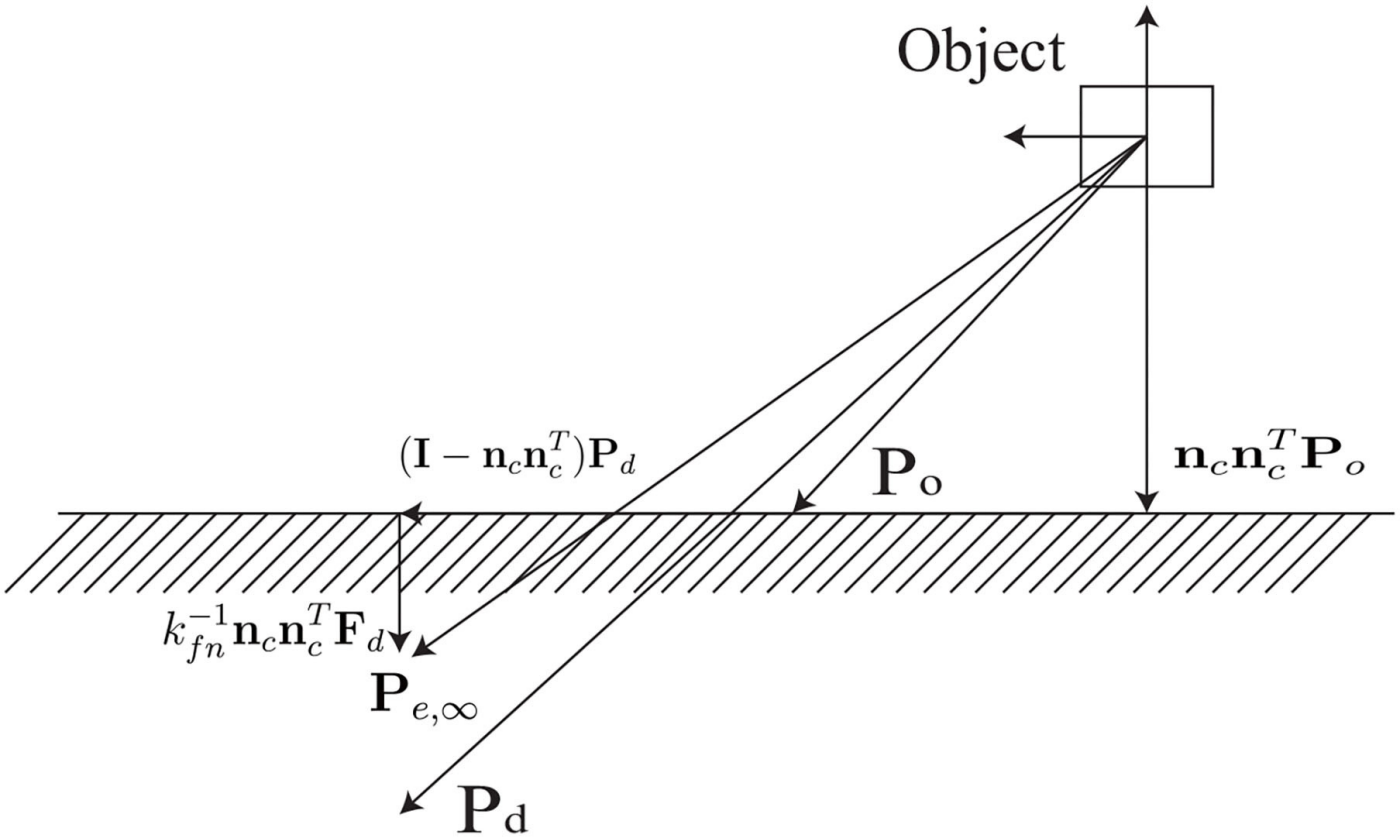
Model of the equilibrium position with force and position control.

We can see from Figure 2, when a desired position **P**_*d*_ is given, the corresponding **P**_*e*_ can be obtained. **P**_*e*_ is different from **P**_*d*_ because of a vector normal to the contact plane and to guarantee **F**_∞_ = **F**_*d*_. Since the equilibrium model is described in the Cartesian space, with the help of (1) and (5), the motion control realized in task space can be projected to image feature space and the corresponding equilibrium position in vision system can be obtained with force and visual servoing.

For the system (12), achieved with the following linear behavior of the system error :


(16) $$\begin{array}{l} {\hat{\text{L}}}_{s}^{+}\left({\ddot{\text{e}}}_{s}+{\text{K}}_{v}{\dot{\text{e}}}_{\text{s}}+{\text{K}}_{p}{\text{e}}_{\text{s}}\right)+{\text{K}}_{f}{\text{e}}_{f}+{\text{K}}_{i}\int_{0}^{t}{\text{e}}_{f}dt=0 \end{array}$$

where **K**_*v*_ = *K*_*v*_**I**, **K**_*p*_ = *K*_*p*_**I**, **K**_*v*_ = *K*_*f*_**I**, **K**_*i*_ = *K*_*i*_**I**.

We project (16) to ∑_*c*_ and get


(17) $$\begin{array}{l} {\hat{\text{L}}}_{s}^{+}\left({\ddot{\text{e}}}_{st}+{\text{K}}_{v}{\dot{\text{e}}}_{\text{s}\text{t}}+{\text{K}}_{p}{\text{e}}_{\text{s}\text{t}}\right)=0 \end{array}$$

(18) $$\begin{array}{l} {\hat{\text{L}}}_{s}^{+}\left({\ddot{\text{e}}}_{ns}+{\text{K}}_{v}{\dot{\text{e}}}_{\text{n}\text{s}}+{\text{K}}_{p}{\text{e}}_{\text{n}\text{s}}\right)+{\text{K}}_{f}{\text{e}}_{nf}+{\text{K}}_{i}\int_{0}^{t}{\text{e}}_{nf}dt=0 \end{array}$$

The Equation (17) gives the dynamic of the components of the position error on the contact plane. Its stability can be obtained for any *K*_*v*_,*K*_*p*_ > 0. The Equation (18) gives the components of the position error and the force error in normal direction. Considering the contact model (14), we have:


(19) $$\begin{array}{l} {\hat{\text{L}}}_{s}^{+}\left({\text{e}}_{\text{n}\text{s}}\right)=k_{f,n}^{-1}{\text{e}}_{nf}-{\text{d}}_{n} \end{array}$$

in which


(20) $$\begin{array}{l} {\text{d}}_{n}=k_{f,n}^{-1}{\text{F}}_{dn}-{\text{P}}_{dn}+{\text{P}}_{on} \end{array}$$

Then the Equation (18) can be transformed to


(21) $$\begin{array}{l} k_{n,f}^{-1}{\ddot{\text{e}}}_{nf}+k_{n,f}^{-1}{\text{K}}_{v}{\dot{\text{e}}}_{nf}+k_{n,f}^{-1}{\text{K}}_{p}{\text{e}}_{nf}+{\text{K}}_{f}{\text{e}}_{nf}+{\text{K}}_{i}\int_{0}^{t}{\text{e}}_{nf}dt=\phi_{n} \end{array}$$

where


(22) $$\begin{array}{l} \phi_{n}={\ddot{\text{d}}}_{n}+{\text{K}}_{v}{\dot{\text{d}}}_{n}+{\text{K}}_{p}{\text{d}}_{n} \end{array}$$

Equation (22) is a third-order system and it can be reached only if the gains satisfy the following condition:


(23) $$\begin{array}{l} K_{i}<K_{v}\left(k_{n,f}^{-1}K_{p}+K_{f}\right) \end{array}$$

## 4. Description of the Collaborative Machine: Delta Robot

**Figure 3:**
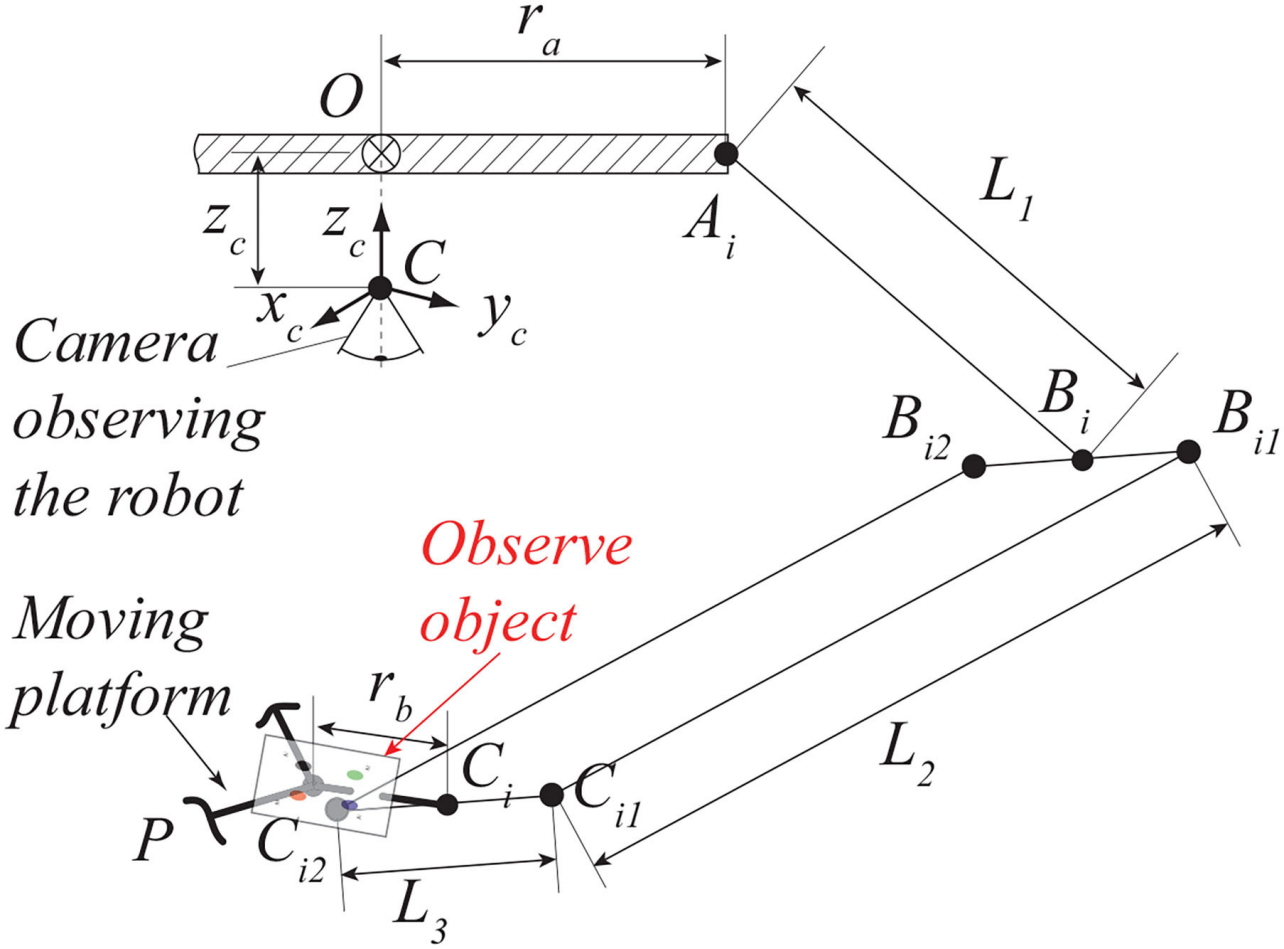
The structure of a Delta robot (one kinematic chain).

Delta robot is a well-known parallel robot with three degrees of freedom. The moving platform can only translate along the three axes of the space with respect to the fixed base. The symmetrical architecture of the Delta robot is given in Figure 3. The moving platform is connected to the base with three identical kinematic chains. Each chain is actuated by the revolute motor located at *A*_*i*_(*i* = 1, 2, 3) in the base. A spatial parallelogram *B*_*i*1_*B*_*i*2_*C*_*i*2_*C*_*i*1_ (*i* = 1, 2, 3) is used to connect the active link *A*_*i*_*B*_*i*_ (*i* = 1, 2, 3) and the platform. The triangles *A*_1_*A*_2_*A*_3_ and *C*_1_*C*_2_*C*_3_ are equilateral, the links *A*_*i*_*B*_*i*_ (*i* = 1, 2, 3) are moving in vertical planes containing *OA*_*i*_, the lengths for each link belonging to the different legs are the same: $||\vec{A_{i}B_{i}}||=L_{1}$, $||\vec{B_{i}C_{i}}||=L_{2}$, $||\vec{B_{i1}B_{i2}}||=||\vec{C_{i1}C_{i2}}||=L_{3}$ for (*i* = 1, 2, 3).

Since the Delta robot can only translate along the three axis in space, we only consider the terms along the end-effector DOF, and the Coriolis and centrifugal terms are not taken into account. The dynamics of the moving platform projected to the task space are then reduced to:


(24) $$\begin{array}{l} {\text{F}}_{p}=\text{M}\ddot{\chi }-{\text{G}}_{p}+\text{F} \end{array}$$

where **M** is the mass matrix of the platform and **G**_*p*_ is the gravity effect of the moving platform of the Delta robot. **F** is the external force applied to the platform because of the interaction with the environment.

**Figure 4:**
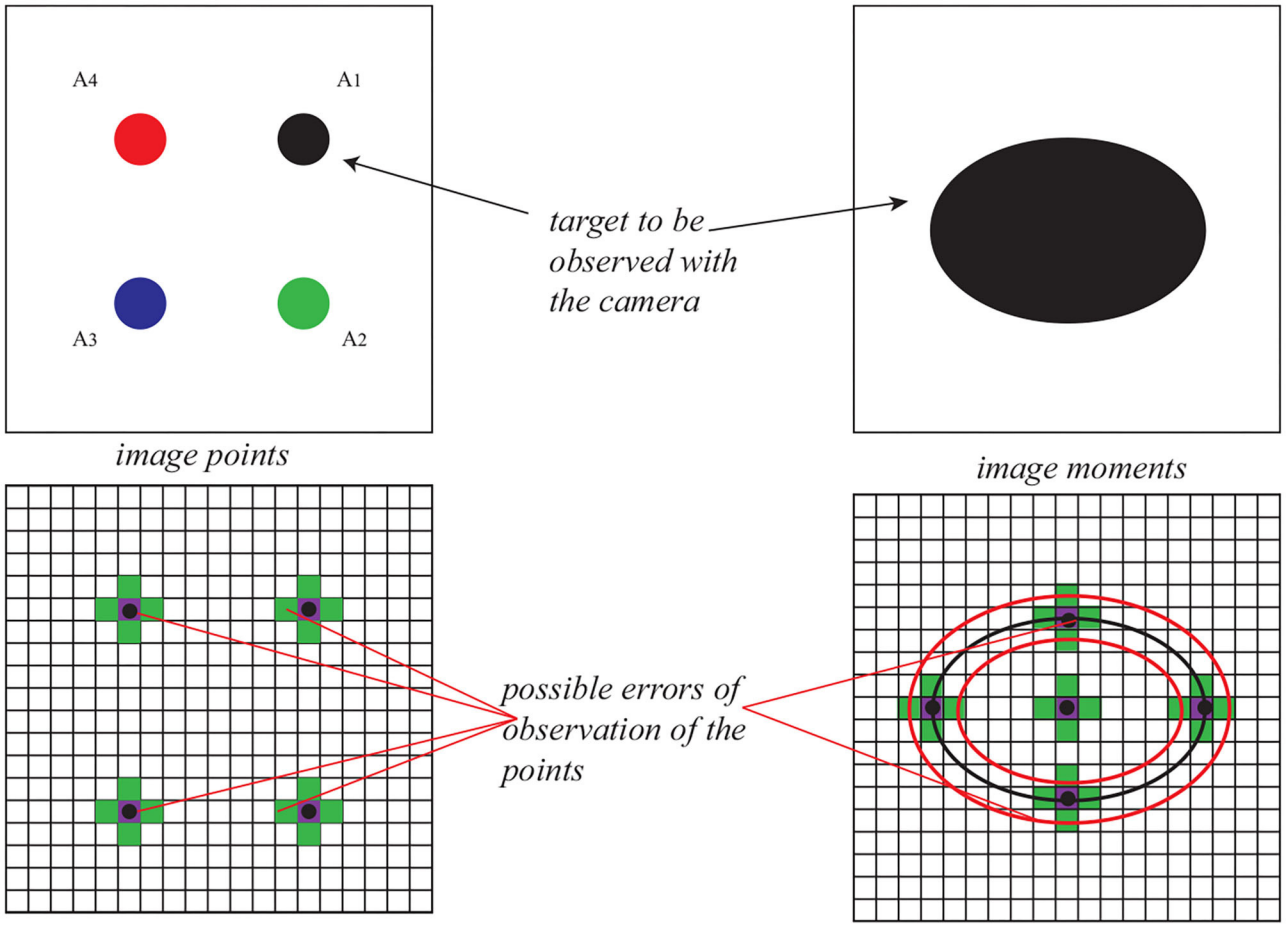
The possible camera observation error for image points and image moments.

To fully control the three DOF of the Delta robot and avoid the global minima at the same time (Zhu et al., 2020), the target to be observed is designed to be four points in one plane (for image points) and an ellipse (for image moments). Then, the image feature **s** is defined as $\text{s}={\left[x_{1},y_{1},x_{2},y_{2},x_{3},y_{3},x_{4},y_{4}\right]}^{T}$ for image points visual servoing and $\text{s}={\left[x_{g},y_{g},a\right]}^{T}$ for image moment visual servoing (*x*_*g*_, *y*_*g*_ the center of the gravity of the target and *a* the area of the target; see Figure 4). The interaction matrix **L**_*s*_ corresponding to the 4 image points can be obtained by stacking (7). For image moment visual servoing, the expression of the corresponding interaction matrix is as follows:


(25) $$\begin{array}{l} {\text{L}}_{s}=\left[\begin{array}{ccc} -C & 0 & Cx_{g}\\ 0 & -C & Cy_{g}\\ 0 & 0 & 2aC \end{array}\right] \end{array}$$

where $c=\frac{1}{Z}$, and *Z* being the depth between the camera and the object.

## 5. Simulation

### 5.1. Specification of the Simulation Environment

To test the parallel IBVS/force controller performance, co-simulations are performed in a connected Simulink-ADAMS environment. The Delta robot mechanical model is created in the software ADAMS with the geometric and dynamic parameters in Table 1, and the pinhole model of the camera and the controller is developed with Matlab/Simulink. To simulate the manufacturing and assembly error in reality, an uncertainty of 10% is added to the inertia parameters of the moving platform of the Delta robot and a 50 μm error is added to the geometric parameters as it was done in Bellakehal et al. (2011). The planar target is assumed to be attached to the moving platform. The contact surface of the target object is parallel to the base of the Delta robot. Then, there exerts a perpendicular force onto the surface of the moving platform when it is contacted with the target object surface. The interaction surface is defined as a spring and the model can be written as follows:


(26) $$\begin{array}{l} \text{F}={\text{K}}_{e}\Delta \chi \end{array}$$

The stiffness of the spring model takes the value of 10^4^ N/m.

**Table 1:** Geometric and dynamic parameters of the Delta robot.

**Delta robot**	
**Parameters**	**Value**
Radius of the base *r*_*a*_ (m)	0.1
Radius of the moving platform *r*_*b*_ m	0.05
Length of *L*_1_ (m)	0.1
Length of *L*_2_ (m)	0.25
Length of *L*_3_ (m)	0.04
Mass of moving platform (kg)	1.94

During this simulation, the force sensor is fixed to the upper surface of the end-effector of the Delta robot and its resolution is $\left[\frac{1}{160}\text{N},\frac{1}{160}\text{N},\frac{1}{160}\text{N}\right]$. A noise of $\pm \frac{1}{160}$ N is added to the force measurement feedback in order to test the robustness of the parallel IBVS/force controller.

### 5.2. Vision System

In this simulation, a single *eye-to-hand* pinhole camera model is considered. This camera is fixed along the *z*_0_ axis of the world frame (see Figure 3) so that the camera can observe the target in a symmetrical way. The resolution of the camera is 1, 920 × 1, 200 pixels and 1 pixel/mm focal length.

For the target to be observed, in image point visual servoing, the coordinates of the four points *A*_1_, *A*_2_, *A*_3_, *A*_4_ are, respectively, (0.3, 0.3,0), (0.3, −0.3,0), (−0.3, −0.3,0), (−0.3, 0.3,0) m, with respect to the moving platform frame. In image moment visual servoing, the target is a circumcircle of *A*_1_, *A*_2_, *A*_3_, *A*_4_.

It has been proven in Zhu et al. (2020) that the positioning error of visual servoing comes from the camera observation error. Therefore, in this case, the value of the camera observation noise is set to be ±0.1 pix, which is a typical noise for cameras roughly calibrated (Bellakehal et al., 2011, see Figure 4). In Zhu et al. (2020), it has been detaily presented on how to add noise when observing an ellipse using image moment visual servoing (Figure 4).

### 5.3. Simulation Results

**Figure 5:**
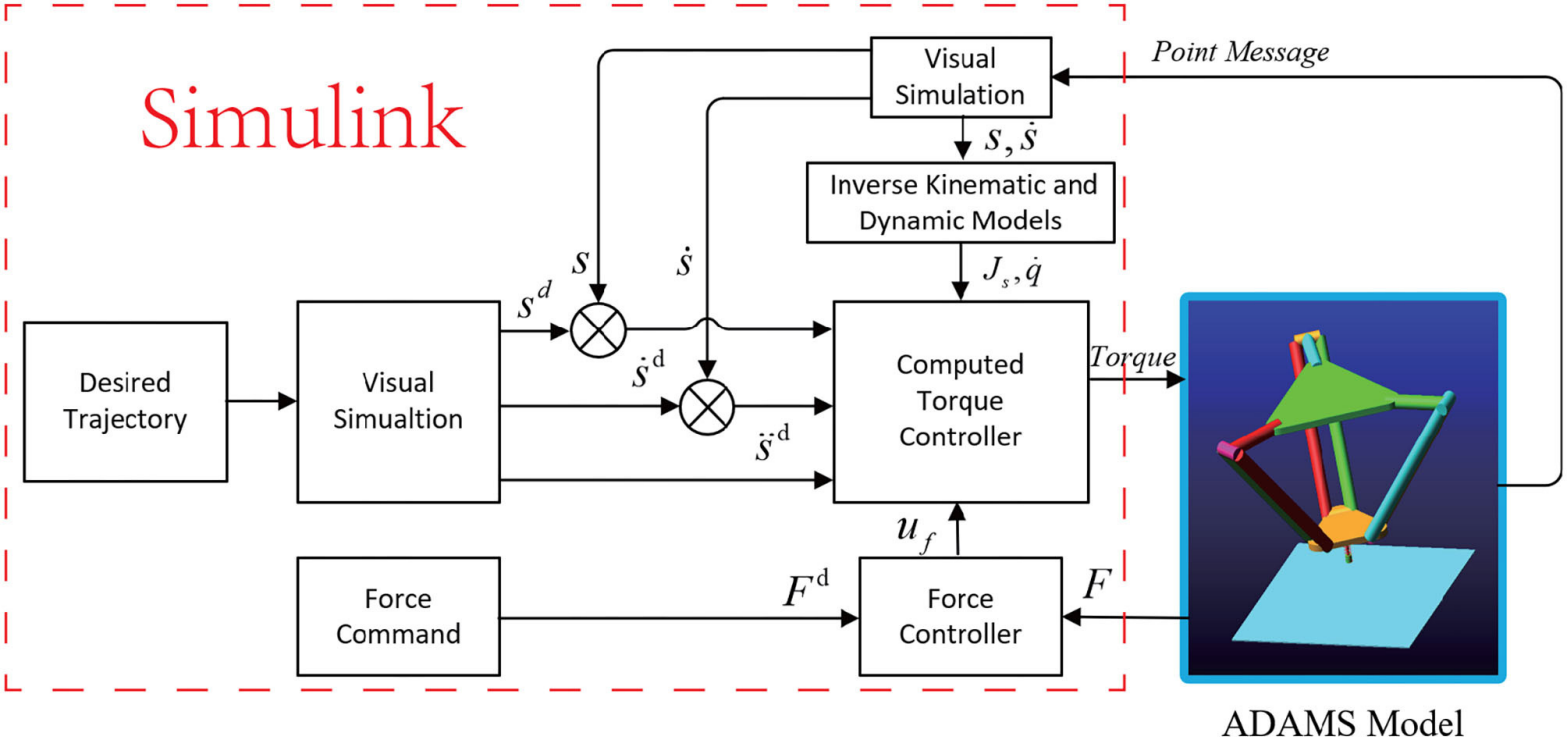
The scheme of the co-simulation between ADAMS and Simulink.

In this section, the parallel IBVS/force control scheme is applied to the control of a collaborative Delta robot to track the position trajectory and force command. For the controller based on image points, we extracted the coordinates of the image points. For the controller based on the image moment, we extracted the coordinates of the centroid point of the observed ellipse and the two points at the extremity of its minimal and maximal radii. Based on these extracted data, we rebuild the image seen by the camera and extract the image feature, use them and the contact force to control the collaborative robot. The scheme of the co-simulation is illustrated in Figure 5.

First we define a position trajectory and a force trajectory. The Delta robot is driven with the corresponding dedicated controller to track these trajectories. The simulations with no noise added to the image plane have been performed to show the effectiveness of the IBVS/force control system. Then, the noise is added to the camera observation and the feedback of the force sensor to show the robustness of the IBVS/force control system. In addition, a comparison between the IBVS/force controller based on image points and image moments has been presented.

The parameters of the controller are given in Table 2 where *K*_*p*_, *K*_*v*_, *K*_*f*_, and *K*_*i*_ are the coefficients of the parallel IBVS/force control in Figure 1.

**Table 2:** Controller parameters.

***K*_*v*_**	***K*_*p*_**	***K*_*i*_**	***K*_*f*_**
5	0.1	600	15.7

**Figure 6:**
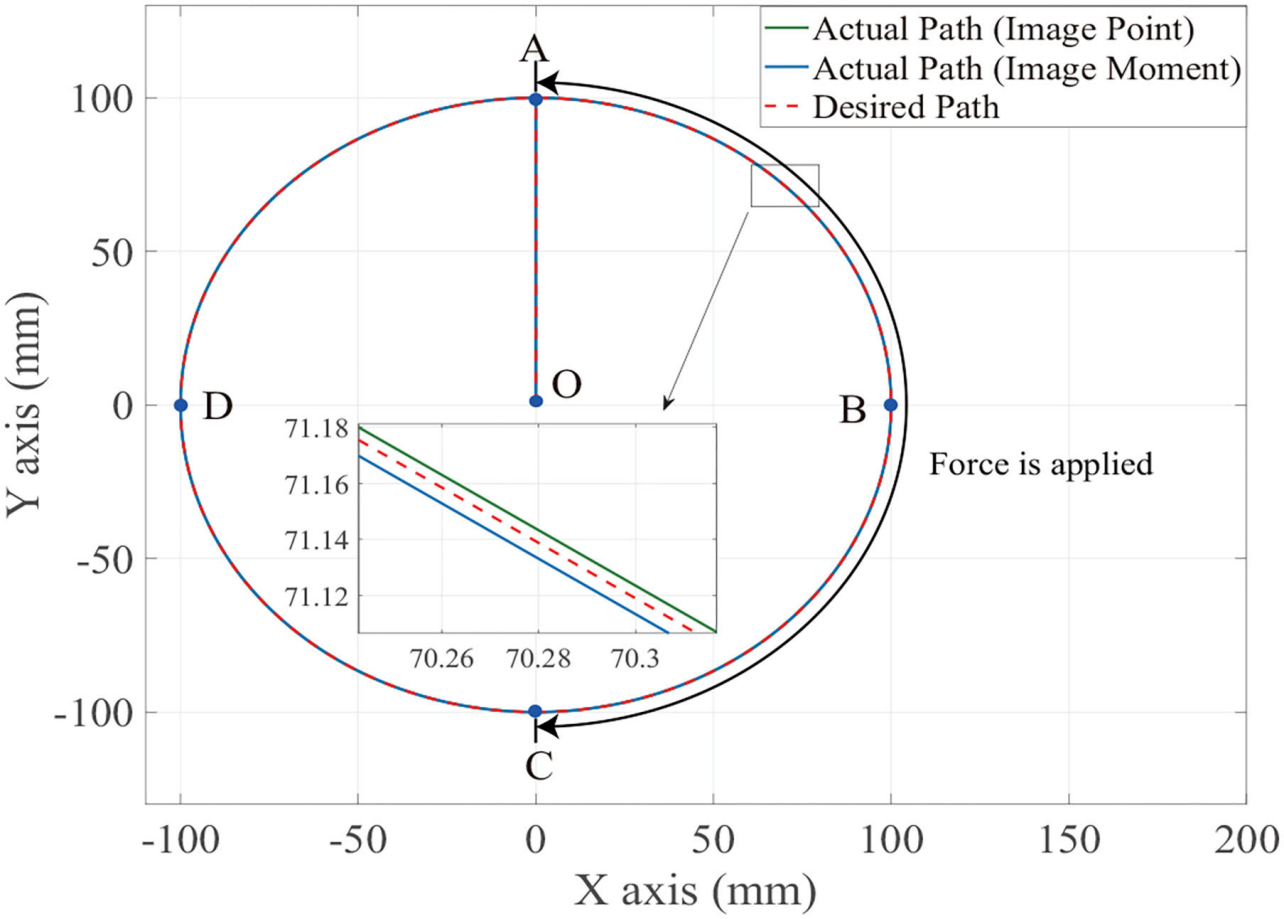
The desired and actual path of moving platform position with no noise in the XY axis.

**Figure 7:**
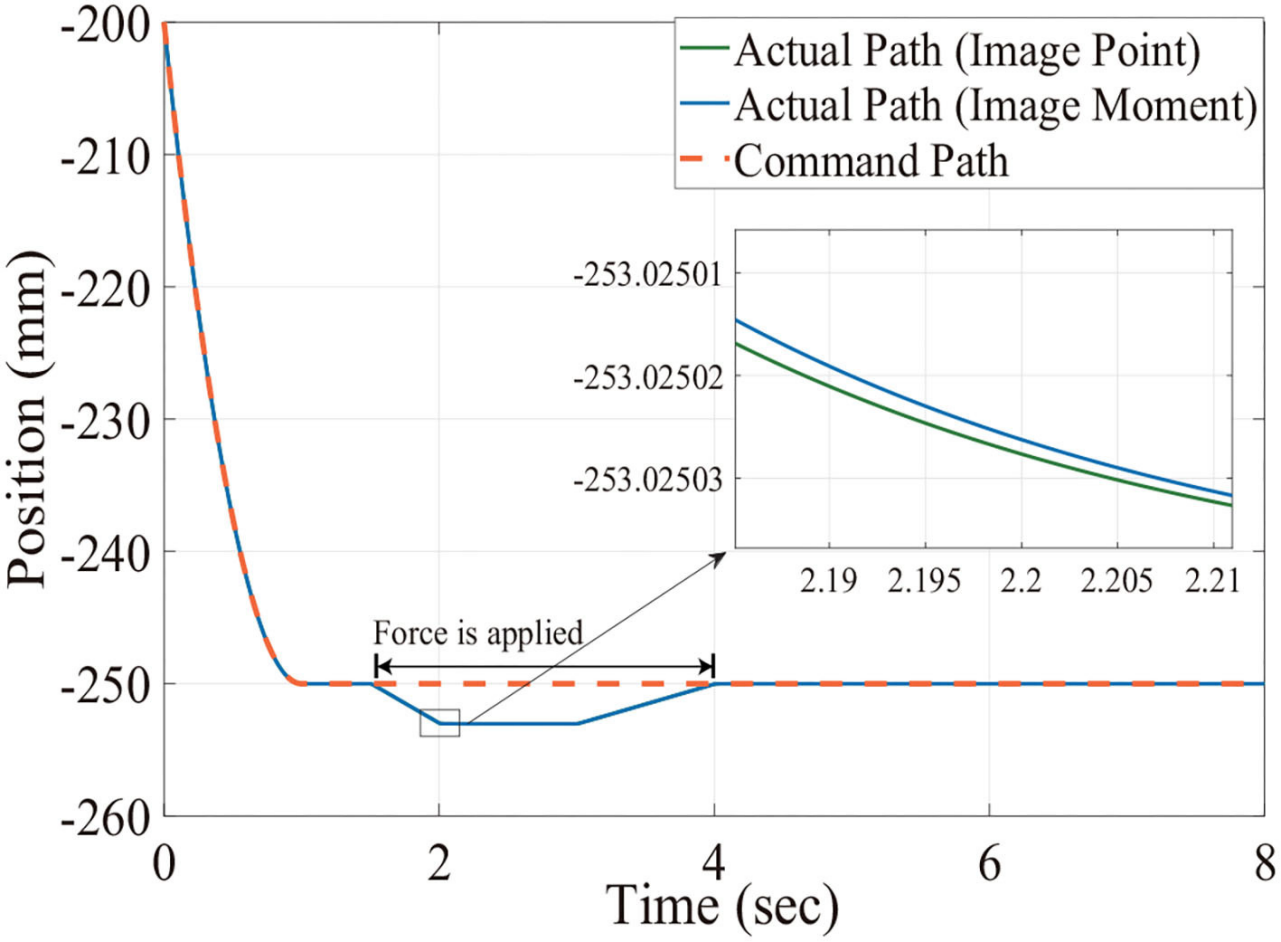
The desired and actual path of moving platform position with no noise in the Z axis.

**Figure 8:**
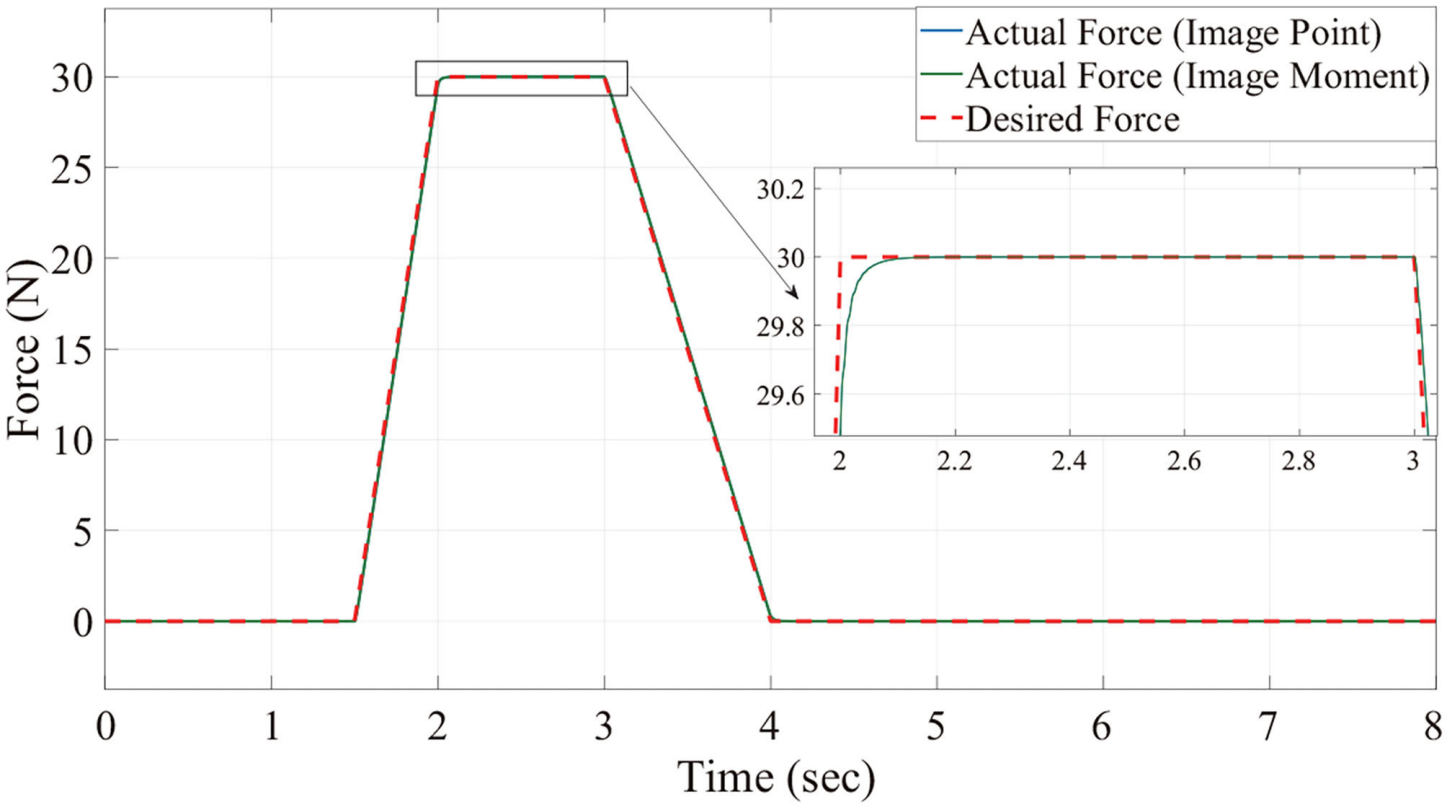
The desired and actual value of force with no noise.

Figures 6, 7 show the position trajectory in x-y plane and z axis, and Figure 8 gives the force command. The moving platform of the Delta robot is driven to move to point A [(0, 100, −250) with respect to the global frame] in 1 s. Then, it is controlled to move clockwise along a circular trajectory in the x-y plane and follow the force command at the same time.

**Figure 9:**
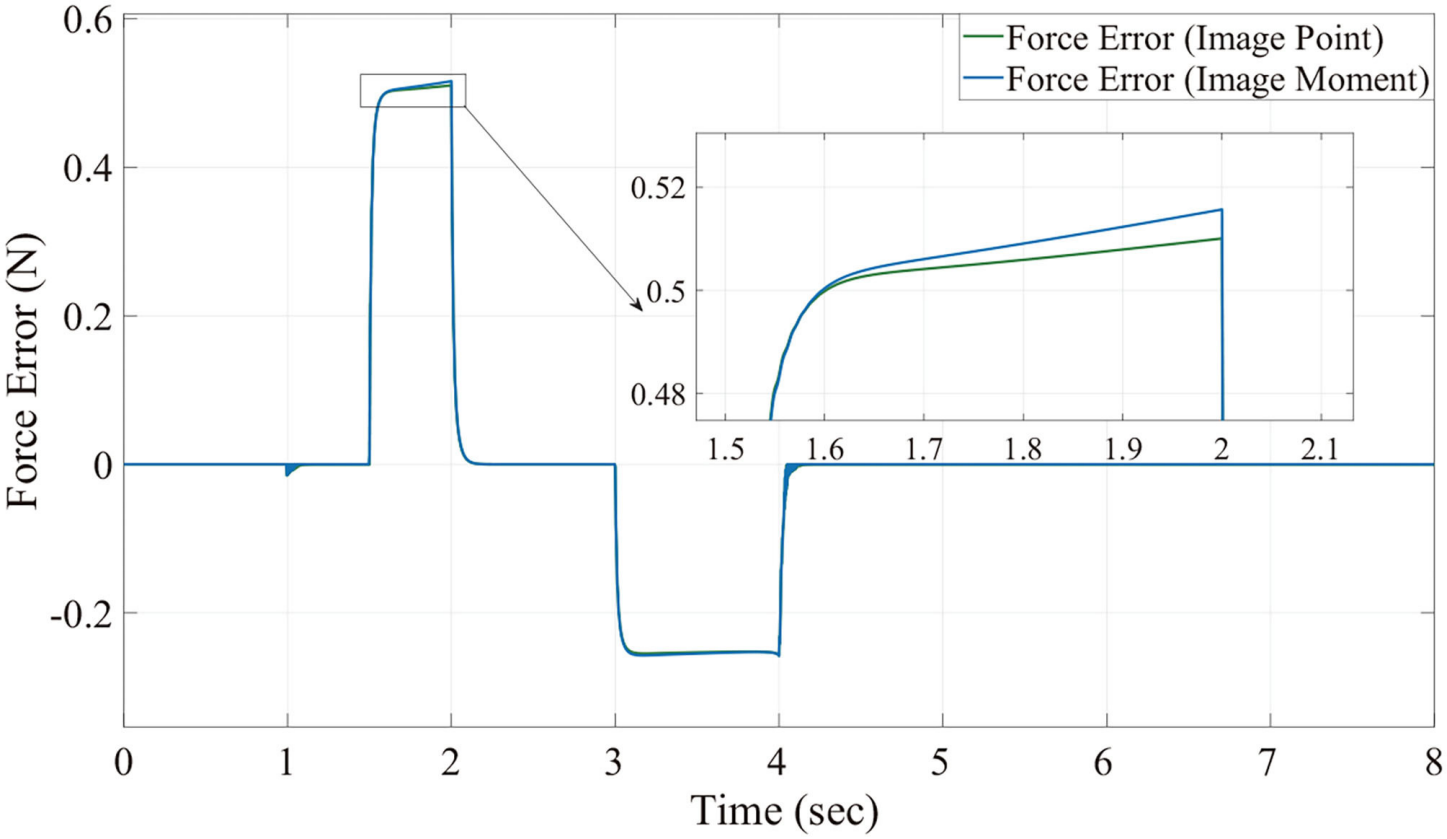
The tracking error of force without noise.

We can see from Figures 6, 7, for both image point visual servoing and image moment visuals servoing, the position errors are within the order of 10^−2^ mm. We see that a residual constant position error exists along the force controlled direction (z axis) after 1.5 s (Figure 7). This error depends on the exerted force and the stiffness of the interaction environment. From the results illustrated in Figures 6, 7, it is obvious that the two kinds of parallel IBVS/force control systems are able to control the position and follow the force trajectory simultaneously. Figures 8, 9 show the force track error and the differences between image point visual servoing/force control and image moment visual servoing/force control are not big enough to draw the conclusion which one is better.

**Figure 10:**
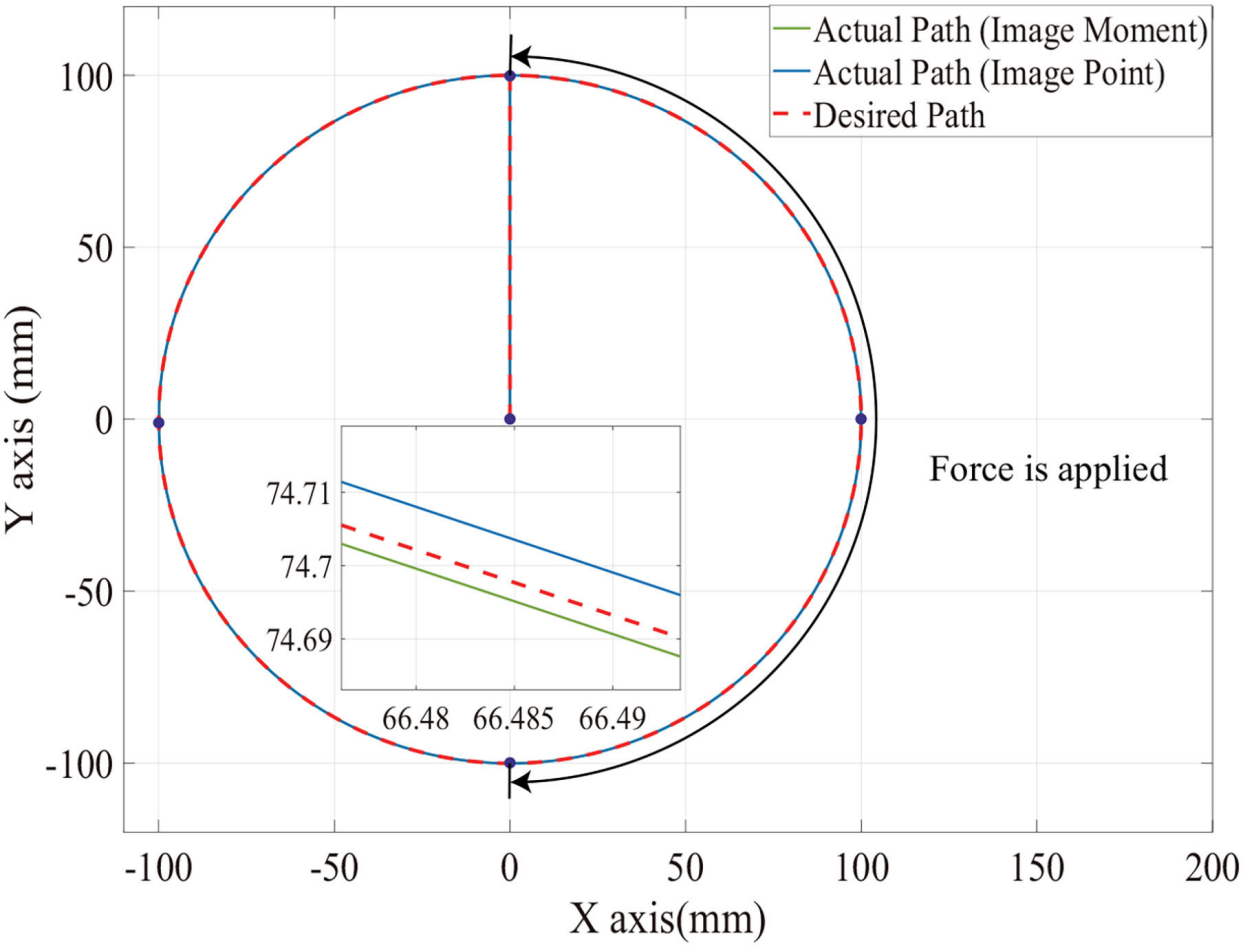
The desired and actual path of moving platform position with noise on XY axis.

Then, in order to test which image feature is more suitable for the parallel IBVS/force control in terms of robustness, the Gaussian noise is added to the camera observation and the feedback of the force sensor. The position trajectory and the force command are the same as we did above. The simulation results are illustrated in Figures 10, **12**, **13**.

**Figure 11:**
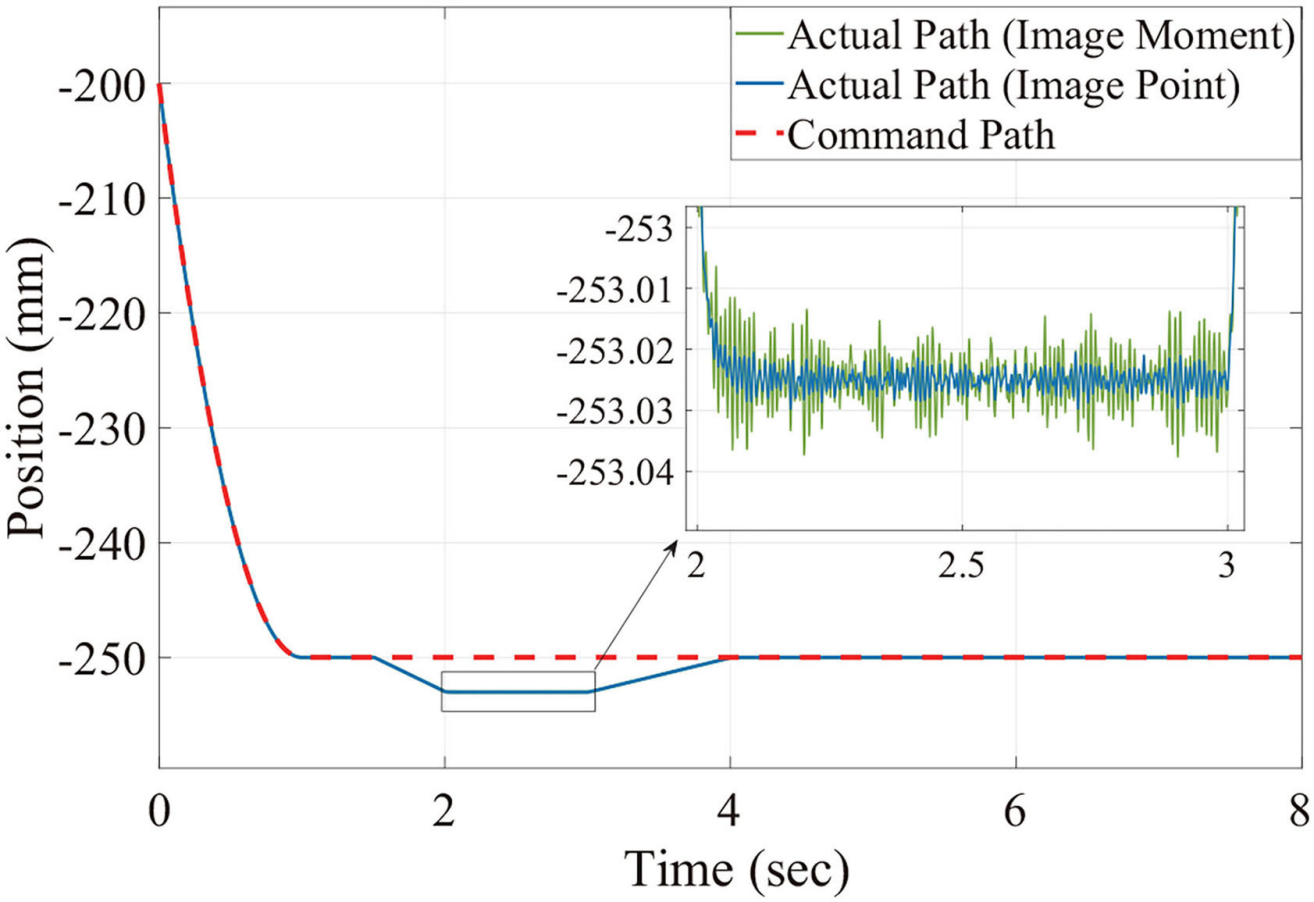
The command and actual value of the moving platform position with noise in the Z axis.

**Figure 12:**
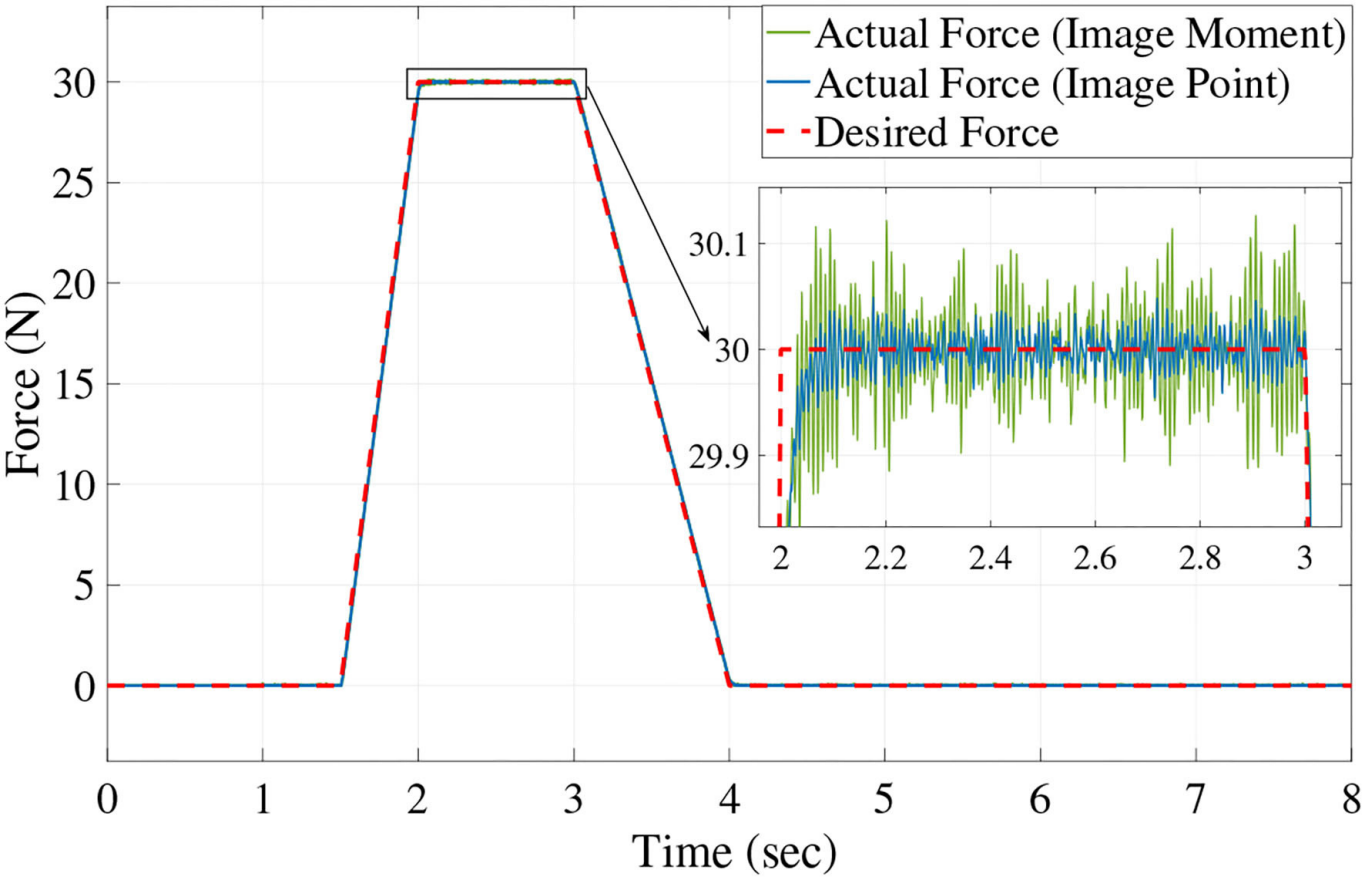
The desired and actual value of force with noise.

**Figure 13:**
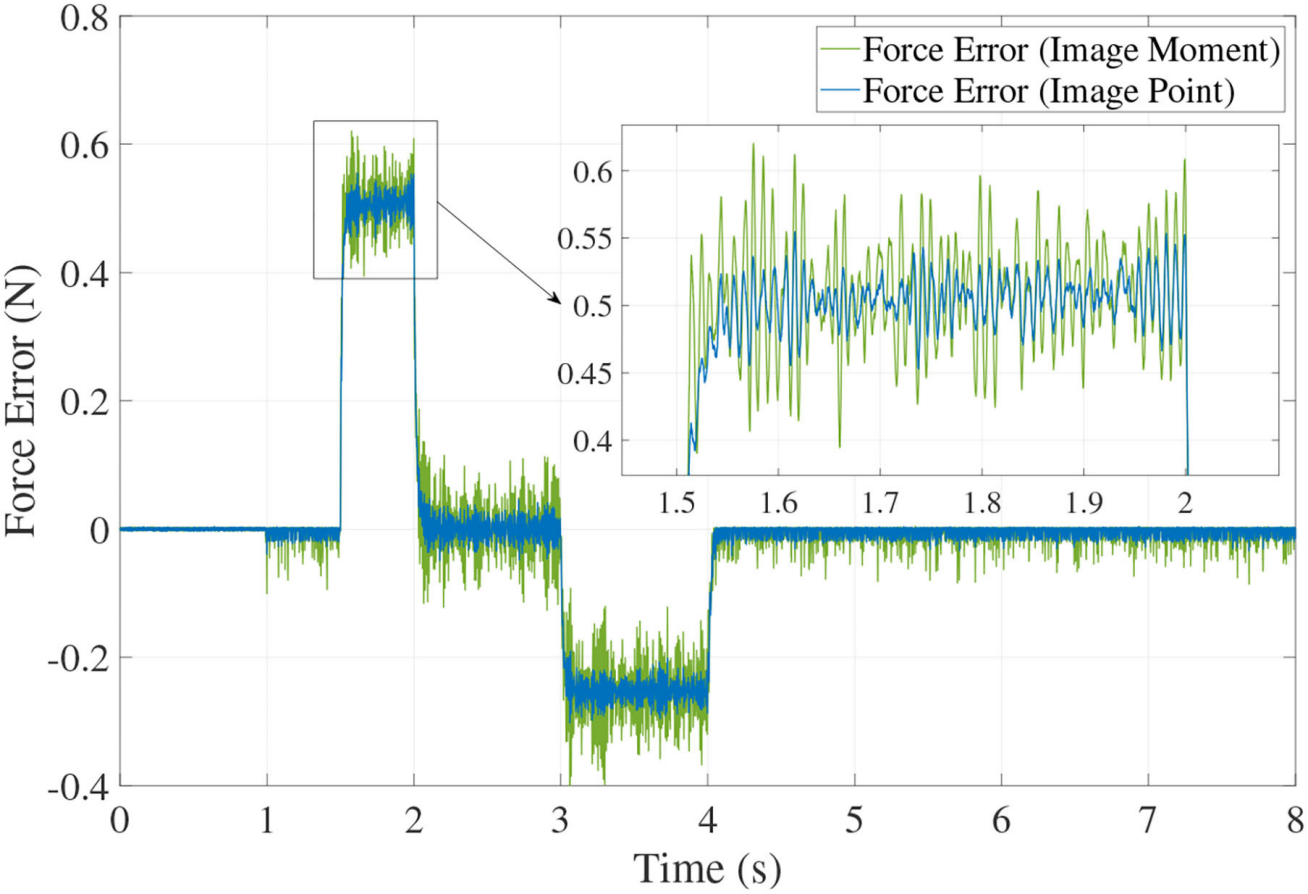
The tracking error of force with noise.

From Figures 11, 12, 13, we see that the robustness of the controller based on the image point is better than the controller based on image moment. When the same noise is added, the position track accuracy and force track accuracy of the image point visual servoing/force controller are two to three times better than those of the image moment visual servoing/force controller.

Compared with previous work by Callegari and Suardi (2003), when the model-based control scheme is used in the position/force control with parallel robots, the force error is more than 3 N while the desired contact force is 10 N (control precision is about 30%). Our simulation results show the overwhelming superiority of parallel IBVS/force upon the model-based position/force control scheme in the case of parallel manipulators. Moreover, compared with the work in Bellakehal et al. (2011), the parallel PBVS/force is applied to the position and force control of parallel manipulators. Based on the premise that the same uncertainty added to the geometric and dynamic parameters of the parallel robots and the noise added to the camera observation and force sensor, the positioning accuracy and orientation accuracy of this controller is 4–10 times better than those in Bellakehal et al. (2011). The force control precision of the parallel IBVS/force controller is about 0.42–1.3% and it is three times better than that of the force controller in Bellakehal et al. (2011), which is about 4%.

## 6. Conclusion

In this paper, a parallel image-based visual servoing/force controller is developed to control the motion of the collaborative robot and follow the contact force command at the same time. This controller has been proposed to generate the output torque signal based on the errors from the image plane and to control the robot by tracking the position trajectory and force command. Two kinds of image features, normalized image points and image moments, are selected to be the feedback of the vision system. The stability of this controller is proven. Simulations are performed on a collaborative Delta robot and the results show that this controller is able to control both contact forces and the motion of the end-effector of the robot at the same time. In addition, the results of the simulation with noise added to the camera observation and force sensor feedback prove that the robustness of the controller based on the image point is better than the controller based on image moment. This proposed parallel IBVS/force controller will be experimentally validated on the collaborative Delta robot prototype in the future.

## Data Availability Statement

The raw data supporting the conclusions of this article will be made available by the authors, without undue reservation.

## Author Contributions

MZ and CH: conceptualization and writing—original draft preparation. MZ and DG: data curation. MZ, ZQ, and WZ: methodology. MZ and WZ: validation. ZQ, WZ, and DG: formal analysis. DG: writing—review and editing and funding acquisition. ZQ: supervision. WZ: project administration. All authors have read and agreed to the published version of the manuscript.

## Funding

This work was supported by the National Natural Science Foundation of China (61803285 and 62001332) and the National Defense Pre-Research Foundation of China (H04W201018).

## Conflict of Interest

The authors declare that the research was conducted in the absence of any commercial or financial relationships that could be construed as a potential conflict of interest.

## Publisher's Note

All claims expressed in this article are solely those of the authors and do not necessarily represent those of their affiliated organizations, or those of the publisher, the editors and the reviewers. Any product that may be evaluated in this article, or claim that may be made by its manufacturer, is not guaranteed or endorsed by the publisher.
